# Does Root-Zone Heating Mitigate the Cold Injury in Coffee Tree (*Coffea arabica*)?

**DOI:** 10.3390/plants14243715

**Published:** 2025-12-05

**Authors:** Mao Suganami, Akira Saeki, Naoto Iwasaki, Daisuke Takata

**Affiliations:** 1 Institute of Fermentation Sciences, Faculty of Food and Agricultural Sciences, Fukushima University, Fukushima 960-1296, Japan; mao.suganami@agri.fukushima-u.ac.jp; 2 Faculty of Food and Agricultural Sciences, Fukushima University, Fukushima 960-1296, Japan; akira-s0709@hotmail.co.jp; 3 School of Agriculture, Meiji University, Kawasaki 214-8571, Japan; iwasaki@meiji.ac.jp

**Keywords:** coffee tree (*Coffea arabica*), cold wind, photosynthesis, root-zone heating, water relation

## Abstract

Cold winter injury is a significant challenge in cultivating tropical trees in temperate regions. The conventional solution involves heating the entire greenhouse to protect the plants; however, this approach is fuel-intensive and costly. This study investigated whether root-zone heating can mitigate cold injury in coffee trees. In the Control, non-heated treatments, leaf relative water content dropped to approximately 70%, leading to wilting, whereas in the Heat treatment, it remained above 90%. In the Control treatment, defoliation progressed, ultimately resulting in more than 50% leaf loss. In contrast, defoliation was reduced by approximately 20% with the Heat treatment. During the cold-treatment period, photosynthesis declined sharply in both the Control and Heat treatments, with CO_2_ assimilation dropping to nearly zero. However, one week after the complete of cold treatment, F_v_/F_m_ recovered to pre-treatment levels, while CO_2_ assimilation and electron transport rates improved to more than 50% of pre-treatment levels in the Heat treatment. These findings indicate that root-zone heating helps prevent leaf wilting and defoliation by maintaining high leaf water content. The surviving leaves recovered their photosynthetic function and were crucial in subsequent biomass production. Thus, root-zone heating is a cost-effective and efficient strategy for cultivating tropical trees in temperate regions.

## 1. Introduction

In recent years, the cultivation of various tropical fruit trees has progressed in Japan [1]. The optimum temperature for the growth of many tropical fruit trees is considered to be between 20 °C and 30 °C [2,3,4]. Similarly, the optimal temperature range for coffee (*Coffea arabica*) is between 18 °C and 22 °C. It is also known that in tropical mountainous regions, the minimum air temperature falls below 10 °C, which is considered the lower limit for coffee cultivation [5].

Coffee cultivation is also beginning to expand in Japan. In Akita Prefecture (in the Tohoku region of Japan), there are cases of coffee cultivation, which background this study. Since tropical fruit trees are sometimes grown outdoors or in unheated greenhouses in Japan, where most regions of the country are in temperate zones (temperatures drop below 0 °C in winter), cold injury has been reported as a significant issue, particularly for avocados, litchis, and papayas during winter [6]. Heating the entire greenhouse to protect the plants from chilling injury is a common practice to prevent cold damage. Coffee production in Japan has not kept up with demand for domestically produced coffee. Therefore, as coffee is a tropical tree, the effects of cold temperatures in Japan during the winter season will be a challenge for the future expansion of coffee cultivation.

Methods using localized heating to maintain a suitable environment have been studied for various ornamental plants, including annuals such as *Saintpaulia* [7], *Ficus benjamina*, *Shefflera arboricola* [8], and *Impatiens walleriana* [9]. A new device capable of controlling the root-zone environment through localized heating and cooling has also been developed [10]. However, research on the relationship between root-zone heating and fruit tree growth remains limited, with only a few studies on olive [11] and papaya [12].

As fruit trees continue to produce fruit for decades, it is essential to prevent cold injury during winter when cultivating tropical fruit trees in temperate regions. Cold injury in tropical fruit trees can be categorized into freeze injury and cold wind damage, both of which are associated with water relations. Freeze injury occurs when tissues cool excessively, leading to cell freezing, influenced by the cytoplasm’s soluble solid content. In contrast, cold wind damage results from dry air at low temperatures, which increases leaf transpiration and causes defoliation. This dehydration occurs due to an imbalance between root water absorption and leaf transpiration [13,14].

In rice, it has been reported that root water absorption is suppressed when the root zone is exposed to low temperatures [15]. Since maintaining root water absorption is crucial for reducing damage from cold winds, heating the plant’s root zone may help sustain root water uptake and suppress leaf wilting.

For evergreen fruit trees, the leaf lifespan is longer than that of many deciduous trees. For example, in citrus fruits grown in temperate regions, leaves formed in spring remain functional not only throughout the year but also after overwintering into the following year [16].

However, until recently, there have been very few examples of coffee trees being cultivated in temperate regions, and no studies have investigated the effects of cold injury on leaf growth and physiological function during or after exposure to low temperatures. Therefore, this study examined whether root-zone heating can mitigate cold injury in coffee trees.

## 2. Results

### 2.1. Temperature Data and Tree Growth

**Figure 1:**
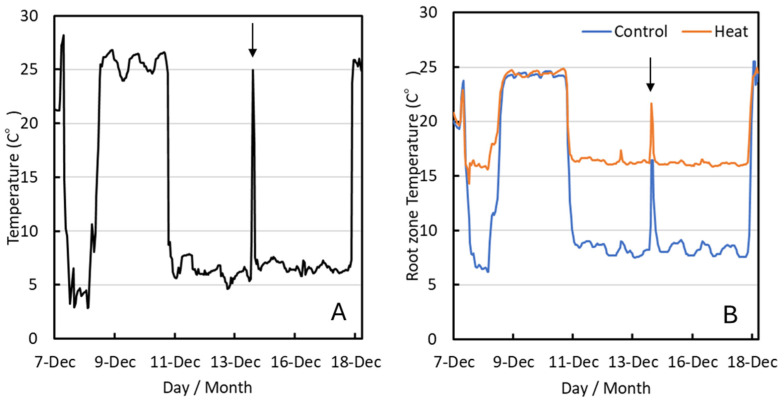
Air temperature (**A**) and root-zone temperature (**B**) in growth chamber during the low-temperature treatment period. The arrow shows when the tree was returned to room temperature for photosynthesis measurement.

In the present experiment, the temperature in the controlled environment growth chamber was set to 5 °C, and as a result, the actual temperature was lowered and maintained at an average of 6.7 °C (±0.2 °C S.E.) during the treatment period from 11 to 18 December, which is below the optimum temperature for coffee growth (Figure 1A). In the Heat treatment, the root-zone temperature was controlled and set to 15 °C, resulting in an average of 16.4 °C (±0.1 °C S.E.), which was maintained near the optimum growth temperature through heating. In contrast, in the Control treatment, the root-zone temperature was not controlled and consequently averaged 8.6 °C (±0.4 °C S.E.), with the soil temperature remaining below the optimum level (Figure 1B).

Air and root-zone temperatures were increased for measurements of stomatal conductance and other parameters (14 December, as indicated by the arrow).

From 8 to 11 December, air and root-zone temperatures increased to room temperature due to a malfunction in the growth chamber.

**Figure 2:**
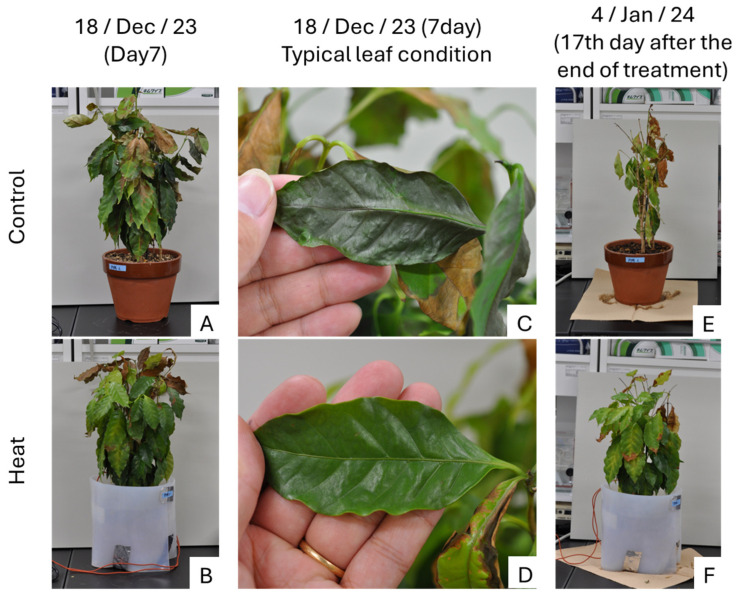
Coffee trees on day 7 of the low-temperature treatment and 17 days after the end of the treatment. (**A**): Control (Day 7); (**B**): Heat (Day 7); (**C**): Leaf of Control (Day 7); (**D**): Leaf of Heat (Day 7); (**E**): Control (17th day after the end of treatment); (**F**): Heat (17th day after the end of treatment); The most damaged trees in both Control and Heat are shown.

The results clearly showed differences in tree appearance, with leaf wilting beginning on the last day of the cold treatment (18 December; Day 7) in the Control treatment (Figure 2A,C). Additionally, rapid leaf drop was observed in two of the three control trees on the 17th day after the treatment ended (4 January; After Day 17) (Figure 2E). In contrast, leaf wilting hardly progresses in the Heat treatment (Figure 2B,D,F).

### 2.2. Result of Stomatal Conductance, SPAD, Defoliation Rate, and Relative Water Content

**Figure A1:**
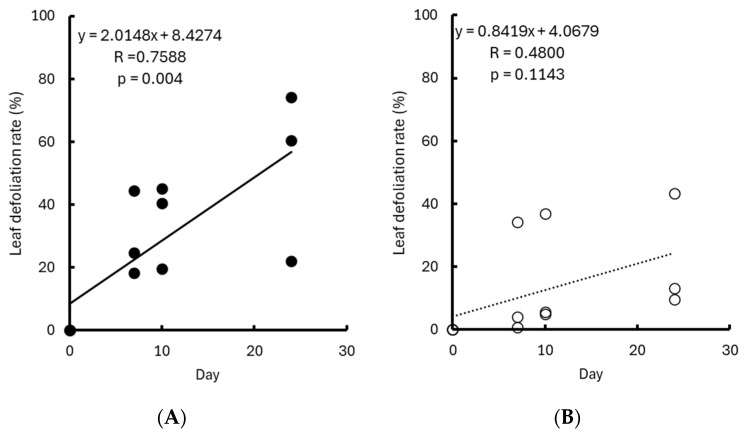
The relationship between the number of days since the start of the low-temperature treatment and the leaf defoliation rate. (**A**): The relationship between the number of days since the start of the low-temperature treatment and the leaf defoliation rate in ‘Control’. (**B**): The relationship between the number of days since the start of the low-temperature treatment and the leaf defoliation rate in ‘Heat’. Data from Day 0, Day 7, after Day 3 (Day 10 after experiment start), and after Day 17 (Day 24 after experiment start) were combined for analysis (*n* = 12).

On Day 7 (18 December), stomatal conductance (g_s_) in the Control treatment (33.6 mmol H_2_O m^−2^ s^−1^) was significantly higher than in the Heat treatment (19.9 mmol H_2_O m^−2^ s^−1^) (*p* = 0.0270, *n* = 3). However, since the CO_2_ assimilation was almost zero on the same measurement day (see below), it is unlikely that the high g_s_ in the control group contributed to CO_2_ assimilation. After Day 17 (4 January), the difference in g_s_ between the Control (31.1 mmol H_2_O m^−2^ s^−1^) and Heat (80.2 mmol H_2_O m^−2^ s^−1^) treatments disappeared (Table 1; *p* = 0.0598, *n* = 3). SPAD values in the Control group were significantly higher than the SPAD values in the Heat group on Day 7 (18 December, *p* = 0.0180, *n* = 3), but the difference disappeared after Day 17 (4 January) (Table 1). Significant differences in RWC were observed during the cold treatment on Day 3 (14 December, *p* = 0.0065, *n* = 3) and 7 (18 December, *p* = 0.0072, *n* = 3). However, after the cold treatment, there was almost no difference between the Control and Heat treatments (Table 1). The defoliation rate was 52.2% in the Control group and 22.0% in the Heat group after Day 17 (Table 1; 4 January *p* = 0.1796, *n* = 3). Because defoliation rates generally increase day by day, a correlation analysis was performed between defoliation rates and the number of days since the start of the low-temperature treatment. A significant positive correlation was observed in the Control group (r = 0.7588, *p* = 0.004, *n* = 12), with defoliation rates increasing as days passed. In contrast, no significant correlation was observed in the Heat treatment group, showing no significant increase in defoliation rate (r = 0.48, *p* = 0.1143, *n* = 12) (Figure A1). Furthermore, using data collected on Day 3 and Day 7 and after Day 3 and Day 17, a linear mixed model was applied to evaluate the effect of root-zone heating on RWC (%). The analysis revealed that root-zone heating significantly increases RWC compared with non-heated Control (β = 13.6, SE = 2.7, *p* = 0.016). The 95% confidence interval for the treatment effect ranged from 4.9 to 22.3, indicating a robust positive influence of root-zone heating on leaf water status.

We also investigated the correlation between g_s_, SPAD, leaf defoliation rate, and RWC data on each measurement day (Day 3 and Day 7 and after Day 3 and Day 17). The results showed a significant negative correlation between g_s_ and RWC on Day 7 (r = 0.9202, *p* = 0.0093, *n* = 6), RWC and SPAD on Day 7 (r = −0.8388, *p* = 0.0370, *n* = 6), g_s_ and leaf defoliation after Day 17 (r = −0.8390, *p* = 0.0368, *n* = 6), and RWC and leaf defoliation after Day 17 (r = −0.8815, *p* = 0.0202, *n* = 6). No significant correlations were observed with the other measurements (Table A1).

### 2.3. Gas Exchange and Chlorophyll Fluorescence

**Figure 3:**
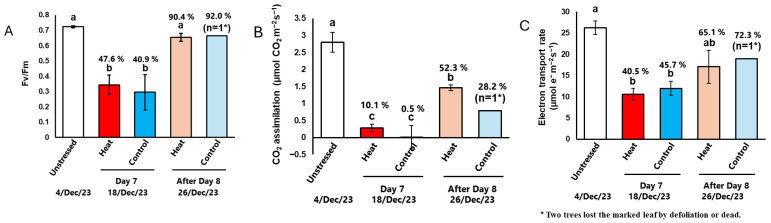
The effects of low-temperature treatment with or without heat on photosynthetic performance in the coffee leaves. Measurement leaves were marked, and the same leaves were measured. (**A**) The maximum quantum yield of PSII, F_v_/F_m_. (**B**) CO_2_ assimilation and (**C**) electron transport rate were measured at 25 °C and an irradiance of 1500 μmol quanta m^–2^ s^–1^, and Ca = 40 Pa. Data are presented as means ± SE (*n* = 3–5, except after Day 8 of Control, where *n* = 1, because two trees lost the marked leaf by defoliation or dead). Statistical analysis was conducted using one-way ANOVA with Holm’s test. Different letters indicate statistical differences among the treatments (*p* < 0.05).

Next, we examined the photosynthetic function of leaves during and after exposure to cold temperatures. The same leaves were marked to track changes in photosynthetic function over time. On the last day of cold treatment (Day 7), F_v_/F_m_, CO_2_ assimilation, and the electron transport rate in both the Heat and Control groups significantly decreased compared to their levels before the cold treatment began (unstressed) (Figure 3, Table S2). Notably, the CO_2_ assimilation rate was close to zero (Figure 3B). By the 8th day after the cold treatment ended (After Day 8), F_v_/F_m_ in the Heat treatment recovered to the same level as in unstressed plants (Figure 3A). Although CO_2_ assimilation and the electron transport rate did not fully recover, they returned 52.3% and 65.1% of the levels observed in unstressed plants, respectively (Figure 3B,C). In contrast, in the Control, the marked leaf fell off in one individual plant and could not be measured. In another plant, although the marked leaf remained, chlorophyll fluorescence could not be measured properly (ETR showed a negative value, as shown in Table A2). Therefore, the leaf was judged to be completely dead. The other one with surviving leaves showed a recovery in photosynthetic function similar to that observed in the Heat treatments. As described above, *n* = 1 for the Control; it is not possible to accurately compare the effect of root-zone heating on the recovery of photosynthetic capacity. However, since defoliation means that photosynthesis becomes zero, the fact that defoliation is prevented by heating can be said to indirectly contribute to the assurance of photosynthetic capacity.

**Figure 4:**
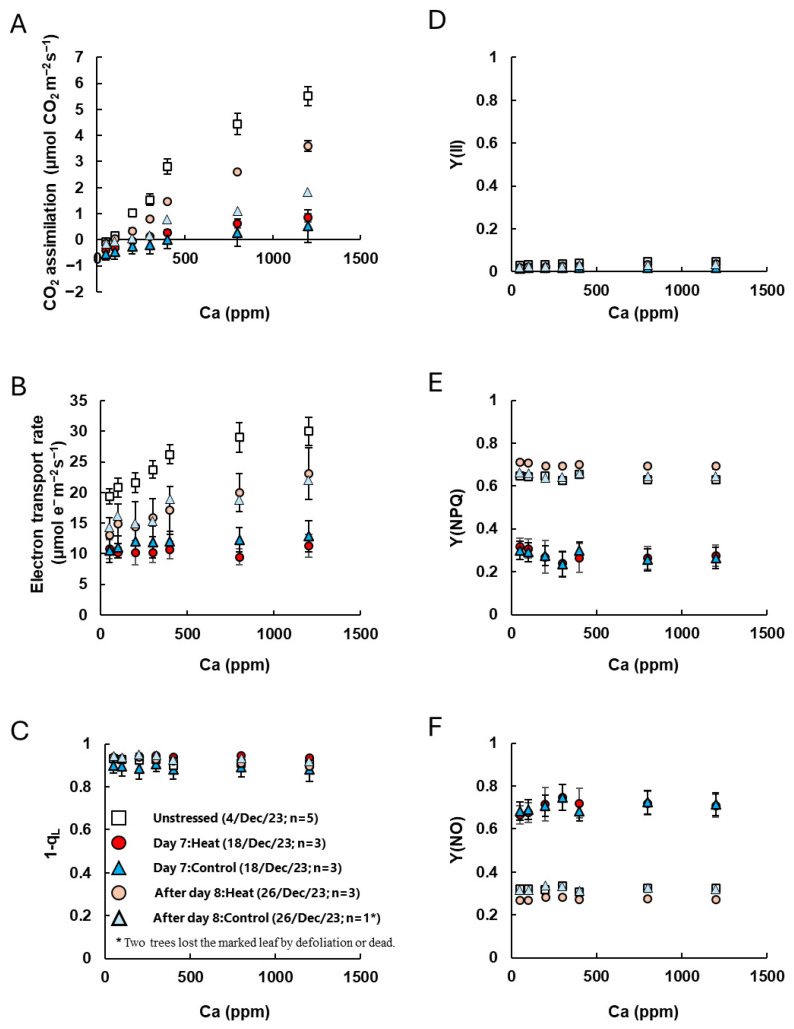
The response of CO_2_ assimilation and chlorophyll fluorescence parameters to changes in CO_2_ concentrations ((**A**): Ca curve). CO_2_ assimilation and chlorophyll fluorescence parameters [(**B**) electron transport rate, (**C**) 1-q_L_, (**D**) quantum yield of PSII, Y(II), (**E**) quantum yield of regulated non-photochemical energy dissipation, Y(NPQ) and (**F**) quantum yield of non-regulated and non-photochemical energy dissipation, Y(NO)] were simultaneously measured at 25 °C, an irradiance of 1500 μmol quanta m^–2^ s^–1^, and different CO_2_ partial pressures. White diamond indicates unstressed leaves before low-temperature treatment. Red and pink circle indicates leaves with heat-treated coffee on Day 7 and after Day 8 of low-temperature treatment, and blue and sky-blue triangles indicate leaves without heat-treated Control coffee on Day 7 and after Day 8 of low-temperature treatment. Measurement leaves were marked, and the same leaves were measured. Data are presented as means ± SE (*n* = 3–5, except after Day 8 of Control, where *n* = 1, because two trees lost the marked leaf by defoliation or dead).

Furthermore, we examined the responses of CO_2_ assimilation and chlorophyll fluorescence to changes in CO_2_ concentration (referred to as [CO_2_] hereafter) and light intensity (Figure 4, Table S3).

**Figure A2:**
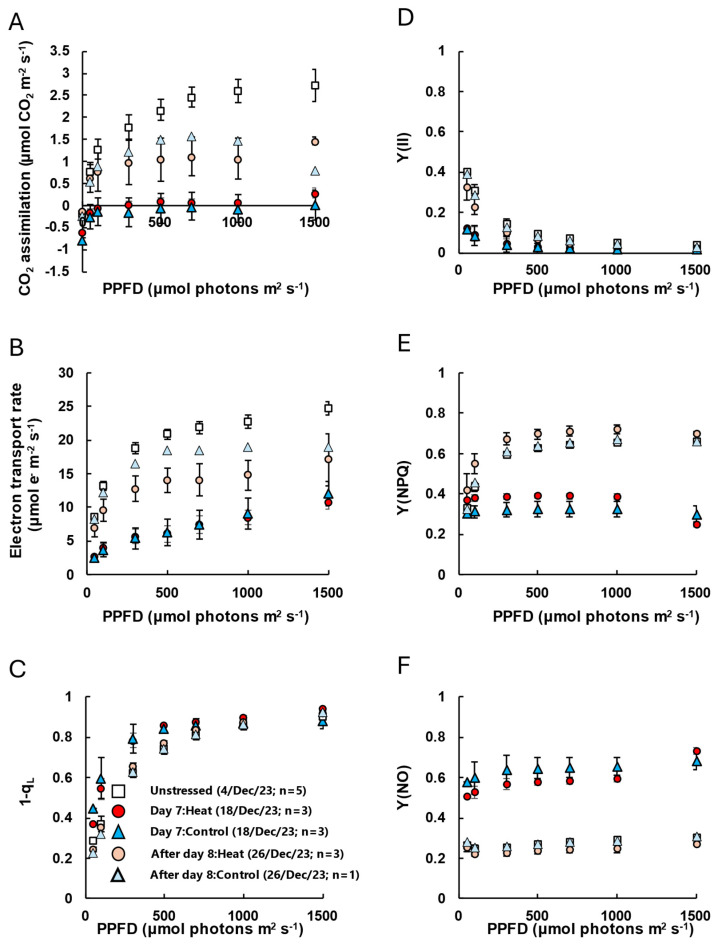
The response of CO_2_ assimilation and chlorophyll fluorescence parameters to changes in light intensity (A:Q curve). (**A**) CO_2_ assimilation and chlorophyll fluorescence parameters [(**B**) electron transport rate, (**C**) 1-q_L_, (**D**) Quantum yield of PSII, Y(II), (**E**) quantum yield of regulated non-photochemical energy dissipation, Y(NPQ) and (**F**) quantum yield of non-regulated and non-photochemical energy dissipation, Y(NO)] were simultaneously measured at 25 °C, Ca = 40 Pa, and different irradiance. White diamond indicates unstressed leaves before low-temperature treatment. Red and pink circle indicates leaves with heat-treated coffee on Day 7 and after Day 8 of low-temperature treatment, and blue and sky-blue triangles indicate leaves without heat-treated control coffee on Day 7 and after Day 8 of low-temperature treatment. Measurement leaves were marked, and the same leaves were measured. Data are presented as means ± SE (*n* = 3–5, except after Day 8 of control, where *n* = 1, because two trees lost the marked leaf by defoliation or dead).

In unstressed plants, CO_2_ assimilation and the electron transport rate increased with rising [CO_2_]. However, on Day 7, there was almost no response to [CO_2_] in both the Heat and Control treatments, and CO_2_ assimilation remained below 1 μmol CO_2_ m^−2^ s^−1^ even at a high CO_2_ level of Ca = 120 Pa (Figure 4A,B). No significant differences were observed in the index for plastoquinone pool reduction (1-q_L_) or the quantum yield of PSII [Y(II)]. However, the quantum yield of regulated non-photochemical energy dissipation [Y(NPQ)] significantly decreased, while the quantum yield of non-regulated non-photochemical energy dissipation increased [Y(NO)] (Figure 4C–F). After Day 8, although CO_2_ assimilation and electron transport rates were slightly lower than those in unstressed plants, Y(NO) and Y(NPQ) were nearly the same as in unstressed plants. We note, however, that the Control group lost two leaves during the cold treatment period, so photosynthesis measurements of Control after Day 8 were based on only one remaining leaf (*n* = 1), making statistical comparisons difficult. Additionally, in the analysis of the response to light intensity, CO_2_ assimilation and electron transport rates were extremely low on Day 7, 1-q_L_ increased, and Y(II) decreased under low-light conditions (Figure A2A–D). Furthermore, Y(NO) was significantly higher in stressed plants than in unstressed plants, while Y(NPQ) did not respond to increasing light intensity and remained low even under high light intensities (Figure A2E,F). After Day 8, Y(NO) had returned to the same level as in unstressed plants, and Y(II) remained low under low-light conditions. This was reflected in an increase in Y(NPQ) and a decrease in 1-q_L_ (Figure A2C–F, Table S4).

## 3. Discussion

Cold winter injuries pose a significant challenge for tropical fruit trees cultivated in temperate regions. These injuries can generally be classified as either cold wind damage or freezing injuries. In this study, we investigated the effectiveness of root-zone heating in mitigating cold wind damage in coffee trees during winter. RWC was higher in the Heat treatment group compared to the Control (Table 1 and Table S1). Furthermore, in the correlation analysis between defoliation rate and the number of days that have passed, a significant positive correlation was observed only in the Control group, and no significant correlation was observed in the Heat treatment group; that is, no significant increase in defoliation rate was observed in the Heat treatment group (Figure A1). Furthermore, the linear mixed model indicates that root-zone heating increased RWC by 13.6% on average. These results indicate that root-zone heating effectively reduced cold wind damage in coffee trees.

We found a significant negative correlation between RWC and leaf defoliation after Day 17 (Table A1). These results suggest that a decrease in RWC induces leaf defoliation. The previous study reported that olive trees experience inhibited root growth during winter, which induces water deficit stress [17]. Therefore, heating the root zone is being considered as a useful method to maintain root growth [11]. Moreover, a previous study on papaya trees demonstrated that root-zone heating helped maintain higher leaf water potential [12]. Cold wind damage is thought to cause leaf drying and wilting due to increased evapotranspiration under dry air conditions associated with low temperatures, which creates an imbalance between water loss and root water uptake [13,14]. A prior study on citrus species found that root water uptake decreased to 17–43% when root temperatures were maintained at 1 °C compared to 15 °C [18]. In the case of maize, it has been reported that root hairs decrease under mild low temperatures and that root elongation is inhibited when low temperatures become more severe [19]. Furthermore, it is known that the absorption of water by roots is promoted when root hairs maintain sufficient contact with the soil [20]. Although no studies examining the relationship between root initiation temperature and water uptake in coffee have been found, the reason for the higher water uptake in citrus is explained as an increase in root elongation due to increased soil temperature [21]. Similarly, a study on aspen trees reported that when soil temperatures were controlled at 5 °C, 10 °C, and 20 °C, root growth accelerated, and water potential values increased in the 20 °C treatment [22].

Furthermore, factors other than root elongation are known to affect water uptake. In maize grown in low-temperature conditions in their root zone, anatomical changes such as root tuberization and alterations in aquaporin activity have been reported to affect water uptake [23]. Although this study did not examine parameters such as root elongation or activity, the results indicate that root-zone heating prevents leaf wilting by sustaining water uptake by the roots.

During the cold treatment period, stomatal conductance in the Heat group was comparable to or lower than that in the Control group (Table 1). Furthermore, photosynthesis was significantly restricted in both the Heat and Control groups during the cold treatment period, with CO_2_ assimilation dropping to nearly zero (Figure 3 and Figure 4, Table S3). The effects of low temperature on photosynthesis include direct damage at the leaf level (e.g., photoinhibition of PSI and PSII and degradation of chlorophyll and photosynthetic proteins [24]) and indirect damage due to reduced water uptake in the root zone (stomatal closure and defoliation). The present study indicates that root-zone heating cannot mitigate direct damage to photosynthesis during the cold treatment period but partially suppresses indirect damage. In addition, the low level of stomatal conductance in the Heat group is considered to be related to cold tolerance. Similar reductions in stomatal conductance as a response to high temperatures have been reported in certain varieties of mango and para rubber trees, where the presence or absence of stomatal closure responses has been linked to cold tolerance in para rubber trees [25,26]. Therefore, the reduction in stomatal conductance observed in the Heat group may also have contributed to the suppression of leaf wilting. In fact, we found a significant negative correlation between RWC and g_s_ on Day 7 (Table A1).

Photosynthesis was severely restricted during the cold treatment period in both the Heat and Control groups, with CO_2_ assimilation dropping to nearly zero (Figure 3 and Figure 4, Table S3). Since increasing [CO_2_] did not significantly enhance CO_2_ assimilation (Figure 4), the decline was likely due to impairment of the photosynthetic system rather than stomatal limitations. During the cold treatment period, F_v_/F_m_ significantly decreased in both treatments, indicating the occurrence of PSII photoinhibition (Figure 3A). Additionally, chlorophyll fluorescence parameters showed a reduction in Y(NPQ) and an increase in Y(NO) (Figure 4 and Figure A2). Under stress conditions that limit photosynthetic activity, mild to moderate stress typically induces NPQ to dissipate excess energy, e.g., [27]. However, under severe stress, an increase in Y(NO) has been observed in various plant species [28,29]. These results suggest that the cold conditions in this experiment caused severe damage to the photosynthetic function of coffee leaves.

One week after cold treatment, CO_2_ assimilation recovered to 52.3% of the level observed in unstressed plants (Figure 3). Furthermore, because leaf wilting had not progressed, the transpiration rates in the Heat treatment leaves began to increase on Day 3 after the temperatures were returned to normal conditions (26/20 °C). The production of new leaves requires time and resources. Previous research has shown that Satsuma Mandarins exhibit little to no photosynthesis during winter but rapidly increase photosynthetic activity in overwintered leaves as temperatures rise [30]. Additionally, recent studies on Scott pine, an evergreen species native to northern regions, have identified a mechanism that enables the overwintering of leaves through sustained quenching induced by chloroplast structural changes during the cold period from winter to early spring [31]. In Scott pine, as in the present study on coffee, a decrease in Y(NPQ) and an increase in Y(NO) were observed. These findings suggest that coffee may also exhibit plasticity, allowing it to substantially reduce photosynthetic activity during cold treatment while rapidly restoring its photosynthetic capacity once optimal temperatures return.

Thus, root-zone heating may help sustain root water uptake activity, potentially preventing leaf wilting and defoliation, and could serve as a low-cost strategy for mitigating winter cold wind damage. Root-zone heating during winter stress might allow plants to resume photosynthesis more quickly and transition to biomass production after overwintering. These preliminary results suggest that coffee, a tropical tree, could potentially be grown at low cost in temperate regions such as Japan using root-zone heating methods.

## 4. Materials and Methods

### 4.1. Plant Materials and Growth Conditions

Coffee (*Coffea arabica*) trees, approximately 40 cm in height, were purchased from nursery (Hanahiroba Co., Ltd., Mie, Kuwana, Japan) and used in the experiment. After being transplanted into approximately 5 L unglazed clay pots using commercially available humus-rich soil (Kojiya Co. Ltd., Ibaraki, Japan), the plants were grown under controlled conditions. The seedling trees were about one year old at the time of the experiment and were healthy with no signs of disease. The trees were grown in a growth chamber (LPH-411SPC, NK-system) under a 15 h day length, with a day/night temperature of 26/20 °C and a light intensity of 200–300 μmol photons m^−2^ s^−1^, for approximately one month. Furthermore, the trees were irrigated to field capacity every day until the end of the experiment, and no fertilizer was applied during experimental period.

The cold treatment was conducted for approximately one week in the same growth chamber, with only the temperature conditions being modified. At the end of the treatment, the plants were returned to their original growth conditions, and their recovery after cold treatment was studied.

### 4.2. Cold Treatment and Root-Zone Heating Treatments

Cold treatment was performed in the same growth chamber as described above. No changes were made to the day length or light conditions; only the temperature in the growth chamber was set at 5 °C. Previous study has shown that 10 °C is considered the lower limit for coffee cultivation [5], so the temperature was set to 5 °C. The actual temperature of growth chamber was recorded by a data logger (TR-72, T&D Co. Ltd., Nagano, Japan) (Figure 1A). In each treatment, three seedlings were used as three single-tree replications.

**Figure A3:**
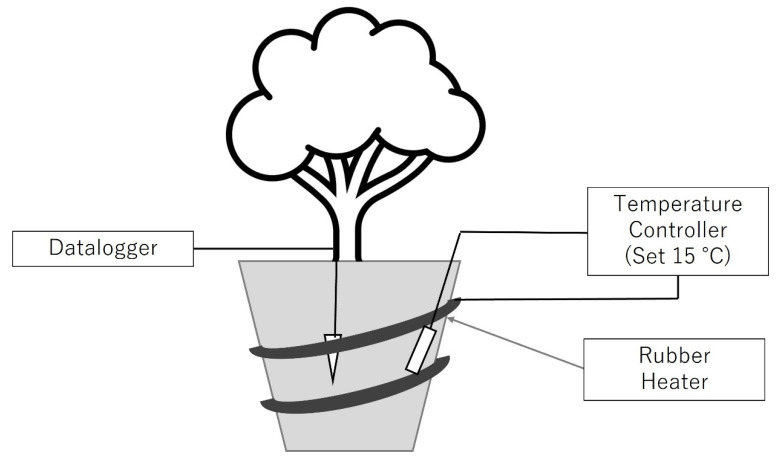
Schematic diagram of the root-zone heating method. A rubber heater was placed around the outside of the pot to heat the root zone. To maintain the optimum temperature, the heater was turned on and off with a temperature controller set at 15 °C. The actual root-zone temperature was recorded using a data logger.

**Figure A4:**
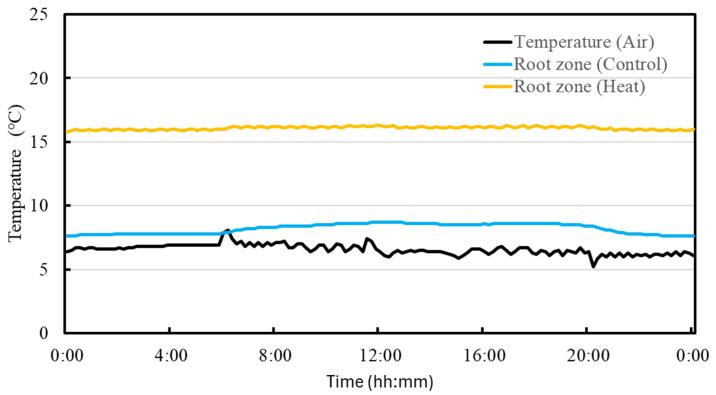
Daily temperature curves for the growth chamber air temperature and root-zone temperature (Control and Heat) (17 December, Day 6).

The experimental setup consisted of two treatments: ‘Heat,’ where the root zone was heated when the soil temperature dropped (Figure A3), and ‘Control,’ where the root zone remained unheated (Figure 1B). Heat treatment was performed by applying silicon rubber heaters around the unglazed clay pots. The rubber heater was controlled using a temperature controller (WT-1001, SHTROL, Xuzhou, China), and the temperature sensor of the controller was placed in the center of the unglazed clay pots. Furthermore, silicon rubber was wrapped around the outside of the unglazed clay pots to keep the root-zone temperature stable (Figure A4).

The actual root-zone temperature was recorded by a data logger (TR71, T&D Co. Ltd., Nagano, Japan). Each treatment had three replicates, with one tree per plot. The treatment was originally scheduled to begin on 7 December 2023. However, due to an equipment malfunction, the temperature of the growth chamber returned to room temperature. As a result, the treatment was resumed on 11 December 2023 and continued for one week until 18 December (Figure 1A). Therefore, the experiment was conducted with 11 December as day 0. Furthermore, during the experiment, the pot positions in the cultivation chamber were randomly rotated.

### 4.3. Measurement Related to Tree Survival

In this experiment, stomatal conductance (g_s_), SPAD values, and relative water content (RWC) were measured on treatment Day 3 (14 December 2023) and treatment Day 7 (18 December) to evaluate the effects of cold treatment and root-zone heating. Additionally, post-treatment measurements were taken on Day 3 (21 December) and Day 17 (4 January 2024) after the end of the treatment. The fully expanded leaves were used for measurement of stomatal conductance, SPAD value, and RWC. Defoliation rates were simultaneously examined via more than three measurements (stomatal conductance, SPAD, and RWC) except for treatment Day 3.

g_s_ was measured using a porometer (SC-1; Meter Group, Inc., Pullman, WA, USA). Three leaves per tree were measured, and the average value was calculated.

SPAD values were measured using a SPAD-502 chlorophyll meter (Minolta Camera Co. Ltd., Osaka, Japan). Similarly to stomatal conductance, SPAD values were obtained from three leaves per tree, and the average value was calculated.

For RWC measurement, fresh weight (FW) was determined by immediately weighing freshly cut leaves from each tree. The weighed leaves were then placed in water, covered, and left to stand for 2 h at room temperature. After this period, excess water was removed with paper towels, and the leaves were immediately weighed to determine the turgor weight (TW). The leaves were subsequently dried in an oven at 70 °C until a constant weight was achieved, and the dry weight (DW) was recorded. RWC was calculated using the following formula: RWC = (FW − DW)/(TW − DW) × 100. Additionally, the difference between TW and FW was calculated as an indicator of leaf water deficit status. RWC was measured on two leaves per tree, and the mean of these measurements was used as the value for each tree.

Defoliation rates were examined at 3 measurement periods, except for the third day after the start of treatment. Defoliation rates were determined by counting the number of leaves on each tree at the start of the experiment and on the measurement day. It is important to note that no new leaves developed during the treatment period or up to 17 days after the treatment ended.

### 4.4. Measurements of Gas Exchange and Chlorophyll Fluorescence

CO_2_ assimilation and chlorophyll fluorescence were simultaneously measured using a portable gas exchange system (LI-6800, LI-COR, Lincoln, NE, USA) equipped with a fluorometer (6800-01/A) on the following dates: before treatment (4 December 2023), 7 days after treatment initiation (18 December 2023), and 8 days after treatment completion (26 December 2023). The measurement conditions were based on previous studies [32,33], while the CO_2_ concentration and light intensity settings were modified. Measurements were conducted at a leaf temperature of 25 °C, a flow rate of 500 μmol s^−1^, a relative humidity of 60–70%, and a leaf-to-air vapor pressure of 1.0–1.2 kPa. Plants were kept in the dark for at least 30 min before measurements were taken. Minimal fluorescence in the dark-adapted state (F_o_) was excited by a weak measuring light (620 nm). A saturating pulse of light (10,000 μmol photons m^−2^ s^−1^, 300 ms) was applied to determine the maximal fluorescence in the dark-adapted state (F_m_) and during illumination with actinic light (F_m’_). The minimum fluorescence (F_o’_) was measured by turning off the actinic light and applying far-red light (720 nm) immediately after the F_m’_ measurement. The steady-state fluorescence level (F_s_) was recorded during actinic light illumination. The maximal quantum yield of PSII was calculated as F_v_/F_m_, and the NPQ was calculated as (F_m_–F_m’_)/F_m’_. The quantum yield of PSII [Y(II)] was calculated as (F_m_–F_s_)/F_m’_ [34]. The parameter for evaluating the redox level of the plastoquinone (PQ) pool, q_L_, was calculated as (F_m’_–F_s_)/(F_m’_–F_o’_) × (F_o’_/F_s_) [35]. The quantum yield of non-regulated and non-photochemical energy dissipation, Y(NO), was calculated as 1/[NPQ + 1 + q_L_(F_m_/F_o_ − 1)], and the quantum yield of regulated non-photochemical energy dissipation, Y(NPQ), was calculated as 1 − Y(II) − Y(NO). The electron transport rate in PSII (ETRII) was calculated as Y(II) × PPFD × α × 0.5. The absorptance (α) was assumed to be 0.84 in this study according to previous study of coffee [36]. Gas exchange parameters were calculated according to the equations of previous study [37]. First, measurements were conducted at seven different CO_2_ concentrations (Ca = 5, 10, 20, 30, 40, 80, and 120 Pa) under actinic light intensity of 1500 μmol photons m^−2^ s^−1^. Next, measurements were conducted at seven different light intensities (PPFD = 50, 100, 300, 500, 700, 1000, and 1500 μmol photons m^−2^ s^−1^) under a Ca of 40 Pa.

### 4.5. Statistical Analysis

The statistical analysis was performed using the Bell Curve for Excel v.4.07 (Social Survey Research Information Co., Ltd., Tokyo, Japan). Differences between the Control and Heat treatments in stomatal conductance, SPAD values, defoliation rates, and relative water content were compared *t*-test after confirming whether homoscedasticity or heteroscedasticity. To evaluate Heat treatment effects across measurement dates, we additionally fitted a linear mixed-effects model with treatment as a fixed effect and measurement date as a random effect. Statistical analyses of F_v_/F_m_, CO_2_ assimilation, and electron transport rate were performed using one-way ANOVA to evaluate group differences. When significant effects were detected, post hoc pairwise comparisons were conducted with two-sample *t*-tests, and the resulting *p*-values were adjusted for multiple comparisons using Holm’s method (*p* < 0.05). Except for Control after Day 8 (26 December 2023), where *n* = 1 due to defoliation or dead, statistical analysis was performed across days and treatments. Pearson’s correlation coefficient was used to test the significance of the correlation, with *p* < 0.05 being considered significant.

## Figures and Tables

**Table 1:** Change in stomatal conductance, SPAD, leaf defoliation rate, and RWC during cold treatment and after treatment.

		g_s_ (mmolm^−2^ s^−1^)	SPAD	Defoliation Rate (%)	RWC (%)
14 December 2023	Control	32.4 ± 4.4 ^Y^	48.4 ± 3.9	- ^Z^	72.1 ± 4.6
(Day 3)	Heat	24.4 ± 2.2	35.6 ± 3.4	-	95.5 ± 0.5
	*p*-value	*p* = 0.2565 ^X^	*p* = 0.1112		*p* = 0.0065
18 December 2023	Control	33.6 ± 2.4	51.3 ± 1.6	29.0 ± 6.4	73.9 ± 3.7
(Day 7)	Heat	19.9 ± 2.2	39.6 ± 1.9	13.0 ± 8.7	94.1 ± 0.9
	*p*-value	*p* = 0.0270	*p* = 0.0180	*p* = 0.2347	*p* = 0.0072
21 December 2023	Control	27.8 ± 5.7	44.2 ± 0.7	35.1 ± 6.4	88.6 ± 3.7
(After Day 3)	Heat	45.6 ± 8.3	35.4 ± 1.8	15.8 ± 8.6	95.1 ± 0.3
	*p*-value	*p* = 0.2247	*p* = 0.0200	*p* = 0.1926	*p* = 0.2647
4 January 2024	Control	31.1 ± 6.5	35.5 ± 5.3	52.2 ± 12.8	89.2 ± 2.2
(After Day 17)	Heat	80.2 ± 13.9	36.0 ± 5.3	22.0 ± 8.8	93.6 ± 0.1
	*p*-value	*p* = 0.0598	*p* = 0.9543	*p* = 0.1796	*p* = 0.2139

^Z^: Defoliation rates on Day 3 were not measured. ^Y^: Data are presented as means ± SE. ^X^: Statistical significance between Control and Heat treatment was evaluated using *t*-test, and the *p*-values are presented (*n* = 3).

## Data Availability

The original contributions presented in this study are included in the article/Supplementary Material. Further inquiries can be directed to the corresponding authors.

## Supplementary Materials

The following supporting information can be downloaded at: https://www.mdpi.com/article/10.3390/plants14243715/s1, Table S1: Change in stomatal conductance, SPAD, leaf defoliation rate, and RWC during cold treatment and after treatment. Values are presented with 95% confidence intervals (95% CI) and expressed also as percentages relative to the control (% of Control); Table S2: The effects of low temperature treatment with or without heat on photosynthetic performance in the coffee leaves. Average, SE, 95% confidence intervals (95% CI) and percentages relative to the control (% of Control) of measurement data are presented (n = 3−5, except for After Day 8 of Control, where n = 1, because two trees lost the marked leaf by defoliation or dead). The same data as in Figure 3 were used; Table S3: The response of CO2 assimilation and chlorophyll fluorescence parameters to changes in CO2 concentrations (A:Ca curve). Average, SE, 95% confidence intervals (95% CI) and percentages relative to the control (% of Control) of measurement data are presented (n = 3−5, except for After Day 8 of Control, where n = 1, because two trees lost the marked leaf by defoliation or dead). The same data as in Figure 4 were used; Table S4: The response of CO2 assimilation and chlorophyll fluorescence parameters to changes in light intensity (A:Q curve). Average, SE, 95% confidence intervals (95% CI) and percentages relative to the control (% of Control) of measurement data are presented (n = 3−5, except for After Day 8 of Control, where n = 1, because two trees lost the marked leaf by defoliation or dead). The same data as in Figure A2 were used.

## Appendix A

**Table A1:** Correlation analysis between g_s_ (stomatal conductance), SPAD, RWC (relative water content), and leaf defoliation rate. Correlation analysis was conducted on each measurement day (*n* = 6).

		g_s_	SPAD	RWC	Leaf Defoliation Rate
14 December 2023 (Day 3)	g_s_	1			
	SPAD	−0.0245 (*p* = 0.9633)	1		
	RWC	−0.5064 (*p* = 0.3059)	−0.5039 (*p* = 0.3081)	1	
	Leaf defoliation rate	−	−	−	−
18 December 2023 (Day 7)	g_s_	1			
	SPAD	0.6065 (*p* = 0.2018)	1		
	RWC	**−0.9202** **(*p* = 0.0093)**	**−0.8388** **(*p* = 0.0370)**	1	
	Leaf defoliation rate	0.7923 (*p* = 0.0602)	0.1715 (*p* = 0.7453)	−0.6247 (*p* = 0.1853)	1
21 December 2023 (After Day 3)	g_s_	1			
	SPAD	−0.5405 (*p* = 0.2682)	1		
	RWC	0.5506 (*p* = 0.2575)	−0.4832 (*p* = 0.3316)	1	
	Leaf defoliation rate	−0.3058 (*p* = 0.5556)	0.2010 (*p* = 0.7026)	−0.7366 (*p* = 0.0949)	1
4 January 2024 (After Day 17)	g_s_	1			
	SPAD	0.0721 (*p* = 0.8920)	1		
	RWC	0.7176 (*p* = 0.1083)	0.4726 (*p* = 0.3439)	1	
	Leaf defoliation rate	**−0.8390** **(*p* = 0.0368)**	0.2027 (*p* = 0.7001)	**−0.8815** **(*p* = 0.0202)**	1

Bold formatting indicates *p* < 0.05.

**Table A2:** Comparison of CO_2_ assimilation and chlorophyll fluorescence parameters between surviving and dead leaves after Day 8 of the Control treatment (*n* = 1, respectively). Chl fluorescence parameters (Fo, Fm, Fv, Fv/Fm) were measured under dark-adapted conditions, and CO_2_ assimilation (μmol CO_2_ m^−2^ s^−1^) and fluorescence parameters (Fs, Fm′, Y(II), and electron transport rate) were measured at 25 °C, an irradiance of 1500 μmol photons m^−2^ s^−1^, and Ca = 40 Pa. While the surviving leaf showed positive values for CO_2_ assimilation, Y(II), and electron transport rate, the dead leaf exhibited negative values for these parameters, indicating that the leaf had completely died.

	CO_2_ Assimilation (μmol CO_2_ m^−2^ s^−1^)	Fo	Fm	Fv	Fv/Fm	Fs	Fm′	Y(II)	Electron Transport Rate (μmol e^−^ m^−2^ s^−1^)
Surviving leaf	1.385	1498.03	4478.42	2980.39	0.666	1344.9	1395.48	0.036	22.92
Dead leaf	−0.048	1872.41	2307.75	435.35	0.19	1115.05	1105.15	−0.009	−5.66
