# Supplementary material for: Does Root-Zone Heating Mitigate the Cold Injury in Coffee Tree (Coffea arabica)?
Source: Plants (Basel). 2025 Dec 5;14(24):3715. doi: 10.3390/plants14243715 (PMC12736433; doi:10.3390/plants14243715)
Supplement: Supplementary file 1 [file plants-14-03715-s001.zip › plants-3963440-supplementary.pdf]

Table S1. Change in stomatal conductance, SPAD, leaf defoliation rate, and RWC during cold treatment and after treatment. Values are presented with 95% confidence intervals (95% CI) and expressed also as percentages relative to the control (% of Control).

		g_s ($mmolm^{-2}s^{-1}$)	SPAD	Defoliation rate (%)	RWC (%)
14/Dec/23 (day3)	Control	32.4 ± 8.7	48.4 ± 7.6	-	72.1 ± 9.0 *
	Heat	24.4 ± 4.4	35.6 ± 4.4	-	95.5 ± 1.1
	% of Control	75.2	73.5	-	132.4
18/Dec/23 (day7)	Control	33.6 ± 4.8 *	51.3 ± 3.1 *	29.0 ± 12.6	73.9 ± 7.2 *
	Heat	19.9 ± 4.3	39.6 ± 3.7	13.0 ± 17.0	94.1 ± 1.8
	% of Control	59.2	77.3	44.8	127.3
21/Dec/23 (after day3)	Control	27.8 ± 11.2	44.2 ± 1.3	35.1 ± 12.5	88.6 ± 7.2
	Heat	45.6 ± 16.3	35.4 ± 3.5	15.8 ± 16.9	95.1 ± 0.7
	% of Control	163.9	80.1	44.9	107.3
4/Jan/24 (after day17)	Control	31.1 ± 12.7	35.5 ± 10.3	52.2 ± 25.0	89.2 ± 4.3
	Heat	80.2 ± 27.3	36.0 ± 10.3	22.0 ± 17.2	93.6 ± 0.3
	% of Control	257.7	101.6	42.2	104.9

[Z]: Defoliation rates on Day 3 were not measured.

[Y]: Data are presented as means ± 95%CI

[X]: * indicate significant difference at p=0.05 between Control and Heat treatment by t-test (n=3).

Table S2. The effects of low temperature treatment with or without heat on photosynthetic performance in the coffee leaves. Average, SE, 95% confidence intervals (95% CI) and percentages relative to the control (% of Control) of measurement data are presented (n=3-5, except for After Day 8 of Control, where n=1, because two trees lost the marked leaf by defoliation or dead). The same data as in Figure 3 were used.

		4-Dec-23	Day 7 (18/Dec/23)		After day 8 (26/Dec/23)	
		Unstressed	Heat	Control	Heat	Control
Fv/Fm	Average	0.723	0.344	0.296	0.654	0.666
	SE	0.007	0.063	0.115	0.025	-
	95% CI	0.014	0.124	0.226	0.050	-
	% of Control	100.0	47.6	40.9	90.4	92.0
CO_2 assimilation	Average	2.800	0.282	0.015	1.463	0.790
	SE	0.288	0.109	0.343	0.083	-
	95% CI	0.565	0.213	0.673	0.162	-
	% of Control	100.0	10.1	0.5	52.3	28.2
Electoron transport rate	Average	26.235	10.618	12.001	17.080	18.977
	SE	1.595	1.397	1.675	3.918	-
	95% CI	3.125	2.739	3.283	7.679	-
	% of Control	100.0	40.5	45.7	65.1	72.3

Table S3. The response of CO_2 assimilation and chlorophyll fluorescence parameters to changes in CO_2 concentrations (A:Ca curve). Average, SE, 95% confidence intervals (95% CI) and percentages relative to the control (% of Control) of measurement data are presented (n=3-5, except for After Day 8 of Control, where n=1, because two trees lost the marked leaf by defoliation or dead). The same data as in Figure 4 were used.

Average

		4-Dec-23 Day 7 (18/Dec/23)			After day 8 (26/Dec/23)	
		Unstressed	Heat	Control	Heat	Control
CO_2 assimilation rate	Ca=5 Pa	-0.073	-0.403	-0.544	-0.126	-0.162
	Ca=10 Pa	0.161	-0.311	-0.459	0.050	-0.075
	Ca=20 Pa	1.016	-0.058	-0.255	0.343	0.043
	Ca=30 Pa	1.538	0.125	-0.207	0.792	0.157
	Ca=40 Pa	2.800	0.282	0.015	1.463	0.790
	Ca=80 Pa	4.447	0.620	0.279	2.605	1.091
	Ca=120 Pa	5.513	0.844	0.526	3.598	1.825
Electron transport rate	Ca=5 Pa	19.341	10.762	10.580	13.091	14.310
	Ca=10 Pa	20.805	10.170	11.073	14.854	16.201
	Ca=20 Pa	21.588	10.212	12.000	14.350	14.997
	Ca=30 Pa	23.755	10.143	11.900	15.851	15.372
	Ca=40 Pa	26.235	10.618	12.001	17.080	18.977
	Ca=80 Pa	28.985	9.479	12.257	19.938	18.829
	Ca=120 Pa	30.003	11.281	12.881	23.095	22.075
1-qL	Ca=5 Pa	0.932	0.928	0.900	0.937	0.944
	Ca=10 Pa	0.928	0.933	0.894	0.929	0.938
	Ca=20 Pa	0.924	0.942	0.886	0.937	0.951
	Ca=30 Pa	0.928	0.946	0.905	0.928	0.948
	Ca=40 Pa	0.901	0.937	0.883	0.920	0.924
	Ca=80 Pa	0.910	0.946	0.892	0.908	0.932
	Ca=120 Pa	0.907	0.935	0.881	0.894	0.918
Y(II)	Ca=5 Pa	0.031	0.017	0.017	0.021	0.023
	Ca=10 Pa	0.033	0.016	0.018	0.023	0.026
	Ca=20 Pa	0.034	0.016	0.019	0.023	0.024
	Ca=30 Pa	0.038	0.016	0.019	0.025	0.024
	Ca=40 Pa	0.041	0.017	0.019	0.027	0.030
	Ca=80 Pa	0.046	0.015	0.019	0.032	0.030
	Ca=120 Pa	0.047	0.018	0.020	0.037	0.035
Y(NPQ)	Ca=5 Pa	0.649	0.318	0.300	0.710	0.663
	Ca=10 Pa	0.645	0.308	0.291	0.707	0.658
	Ca=20 Pa	0.648	0.269	0.274	0.694	0.637
	Ca=30 Pa	0.626	0.238	0.234	0.694	0.641
	Ca=40 Pa	0.653	0.263	0.298	0.700	0.659
	Ca=80 Pa	0.628	0.265	0.256	0.694	0.644
	Ca=120 Pa	0.629	0.275	0.264	0.692	0.644
Y(NO)	Ca=5 Pa	0.321	0.665	0.684	0.269	0.315
	Ca=10 Pa	0.322	0.676	0.691	0.270	0.316
	Ca=20 Pa	0.318	0.715	0.707	0.284	0.339
	Ca=30 Pa	0.336	0.746	0.747	0.281	0.334
	Ca=40 Pa	0.306	0.720	0.683	0.273	0.311
	Ca=80 Pa	0.326	0.720	0.725	0.275	0.326
	Ca=120 Pa	0.324	0.708	0.715	0.271	0.321

Table S3 continued. **SE**

		4-Dec-23	Day 7 (18/Dec/23)		After day 8 (26/Dec/23)	
		Unstressed	Heat	Control	Heat	Control
CO_2 assimilation rate	Ca=5 Pa	0.020	0.095	0.249	0.011	-
	Ca=10 Pa	0.056	0.066	0.292	0.018	-
	Ca=20 Pa	0.132	0.040	0.282	0.114	-
	Ca=30 Pa	0.211	0.012	0.343	0.092	-
	Ca=40 Pa	0.288	0.109	0.343	0.083	-
	Ca=80 Pa	0.412	0.176	0.528	0.071	-
	Ca=120 Pa	0.374	0.152	0.621	0.199	-
Electron transport rate	Ca=5 Pa	1.258	1.537	2.001	2.742	-
	Ca=10 Pa	1.512	0.878	1.780	3.216	-
	Ca=20 Pa	1.571	1.976	2.037	4.106	-
	Ca=30 Pa	1.442	1.592	2.561	3.093	-
	Ca=40 Pa	1.595	1.397	1.675	3.918	-
	Ca=80 Pa	2.426	1.301	1.968	3.097	-
	Ca=120 Pa	2.296	1.907	2.525	4.251	-
1-qL	Ca=5 Pa	0.006	0.003	0.037	0.012	-
	Ca=10 Pa	0.007	0.006	0.043	0.013	-
	Ca=20 Pa	0.005	0.007	0.051	0.018	-
	Ca=30 Pa	0.007	0.001	0.034	0.015	-
	Ca=40 Pa	0.007	0.006	0.049	0.016	-
	Ca=80 Pa	0.011	0.001	0.045	0.016	-
	Ca=120 Pa	0.011	0.004	0.055	0.021	-
Y(II)	Ca=5 Pa	0.002	0.002	0.003	0.004	-
	Ca=10 Pa	0.002	0.001	0.003	0.005	-
	Ca=20 Pa	0.002	0.003	0.003	0.006	-
	Ca=30 Pa	0.002	0.003	0.004	0.005	-
	Ca=40 Pa	0.003	0.002	0.003	0.006	-
	Ca=80 Pa	0.004	0.002	0.003	0.005	-
	Ca=120 Pa	0.004	0.003	0.004	0.007	-
Y(NPQ)	Ca=5 Pa	0.011	0.040	0.041	0.011	-
	Ca=10 Pa	0.011	0.043	0.043	0.012	-
	Ca=20 Pa	0.012	0.076	0.046	0.012	-
	Ca=30 Pa	0.012	0.058	0.058	0.010	-
	Ca=40 Pa	0.011	0.068	0.041	0.008	-
	Ca=80 Pa	0.011	0.053	0.053	0.011	-
	Ca=120 Pa	0.010	0.050	0.051	0.011	-
Y(NO)	Ca=5 Pa	0.011	0.042	0.044	0.009	-
	Ca=10 Pa	0.011	0.045	0.045	0.008	-
	Ca=20 Pa	0.011	0.078	0.050	0.011	-
	Ca=30 Pa	0.013	0.061	0.061	0.011	-
	Ca=40 Pa	0.011	0.069	0.044	0.005	-
	Ca=80 Pa	0.012	0.055	0.055	0.010	-
	Ca=120 Pa	0.012	0.053	0.052	0.009	-

Table S3 continued. 95%CI

		4-Dec-23	Day 7 (18/Dec/23)		After day 8 (26/Dec/23)	
		Unstressed	Heat	Control	Heat	Control
CO_2 assimilation rate	Ca=5 Pa	0.039	0.185	0.488	0.021	-
	Ca=10 Pa	0.110	0.129	0.571	0.036	-
	Ca=20 Pa	0.259	0.078	0.552	0.223	-
	Ca=30 Pa	0.413	0.023	0.673	0.181	-
	Ca=40 Pa	0.565	0.213	0.673	0.162	-
	Ca=80 Pa	0.808	0.346	1.035	0.140	-
	Ca=120 Pa	0.734	0.298	1.217	0.391	-
Electron transport rate	Ca=5 Pa	2.465	3.013	3.921	5.374	-
	Ca=10 Pa	2.963	1.720	3.488	6.303	-
	Ca=20 Pa	3.080	3.873	3.993	8.047	-
	Ca=30 Pa	2.827	3.120	5.019	6.062	-
	Ca=40 Pa	3.125	2.739	3.283	7.679	-
	Ca=80 Pa	4.754	2.549	3.858	6.069	-
	Ca=120 Pa	4.500	3.737	4.949	8.333	-
1-qL	Ca=5 Pa	0.013	0.006	0.073	0.024	-
	Ca=10 Pa	0.013	0.011	0.084	0.025	-
	Ca=20 Pa	0.011	0.014	0.100	0.036	-
	Ca=30 Pa	0.013	0.002	0.067	0.030	-
	Ca=40 Pa	0.014	0.011	0.096	0.032	-
	Ca=80 Pa	0.022	0.003	0.089	0.031	-
	Ca=120 Pa	0.021	0.007	0.108	0.041	-
Y(II)	Ca=5 Pa	0.004	0.005	0.006	0.009	-
	Ca=10 Pa	0.005	0.003	0.006	0.010	-
	Ca=20 Pa	0.005	0.006	0.006	0.013	-
	Ca=30 Pa	0.004	0.005	0.008	0.010	-
	Ca=40 Pa	0.005	0.004	0.005	0.012	-
	Ca=80 Pa	0.008	0.004	0.006	0.010	-
	Ca=120 Pa	0.007	0.006	0.008	0.013	-
Y(NPQ)	Ca=5 Pa	0.021	0.079	0.080	0.022	-
	Ca=10 Pa	0.021	0.085	0.085	0.023	-
	Ca=20 Pa	0.023	0.149	0.091	0.024	-
	Ca=30 Pa	0.024	0.114	0.113	0.020	-
	Ca=40 Pa	0.022	0.132	0.081	0.016	-
	Ca=80 Pa	0.022	0.104	0.103	0.021	-
	Ca=120 Pa	0.021	0.097	0.099	0.022	-
Y(NO)	Ca=5 Pa	0.022	0.083	0.086	0.017	-
	Ca=10 Pa	0.022	0.088	0.089	0.017	-
	Ca=20 Pa	0.021	0.153	0.097	0.021	-
	Ca=30 Pa	0.025	0.119	0.119	0.021	-
	Ca=40 Pa	0.021	0.135	0.086	0.009	-
	Ca=80 Pa	0.024	0.107	0.108	0.019	-
	Ca=120 Pa	0.024	0.103	0.103	0.018	-

Table S3 continued. **% of Control**

		4-Dec-23	Day 7 (18/Dec/23)		After day 8 (26/Dec/23)	
		Unstressed	Heat	Control	Heat	Control
CO_2 assimilation rate	Ca=5 Pa	100.0	548.7	740.5	171.9	220.1
	Ca=10 Pa	100.0	-193.0	-284.6	31.2	-46.8
	Ca=20 Pa	100.0	-5.7	-25.1	33.7	4.3
	Ca=30 Pa	100.0	8.2	-13.4	51.5	10.2
	Ca=40 Pa	100.0	10.1	0.5	52.3	28.2
	Ca=80 Pa	100.0	13.9	6.3	58.6	24.5
	Ca=120 Pa	100.0	15.3	9.5	65.3	33.1
Electron transport rate	Ca=5 Pa	100.0	55.6	54.7	67.7	74.0
	Ca=10 Pa	100.0	48.9	53.2	71.4	77.9
	Ca=20 Pa	100.0	47.3	55.6	66.5	69.5
	Ca=30 Pa	100.0	42.7	50.1	66.7	64.7
	Ca=40 Pa	100.0	40.5	45.7	65.1	72.3
	Ca=80 Pa	100.0	32.7	42.3	68.8	65.0
	Ca=120 Pa	100.0	37.6	42.9	77.0	73.6
1-qL	Ca=5 Pa	100.0	99.6	96.5	100.5	101.3
	Ca=10 Pa	100.0	100.5	96.3	100.1	101.1
	Ca=20 Pa	100.0	102.0	95.9	101.4	102.9
	Ca=30 Pa	100.0	101.9	97.5	99.9	102.1
	Ca=40 Pa	100.0	104.0	98.0	102.1	102.6
	Ca=80 Pa	100.0	103.9	98.0	99.8	102.4
	Ca=120 Pa	100.0	103.1	97.2	98.7	101.3
Y(II)	Ca=5 Pa	100.0	55.6	54.7	67.7	74.0
	Ca=10 Pa	100.0	48.9	53.2	71.4	77.9
	Ca=20 Pa	100.0	47.3	55.6	66.5	69.5
	Ca=30 Pa	100.0	42.7	50.1	66.7	64.7
	Ca=40 Pa	100.0	40.5	45.7	65.1	72.3
	Ca=80 Pa	100.0	32.7	42.3	68.8	64.9
	Ca=120 Pa	100.0	37.6	42.9	77.0	73.6
Y(NPQ)	Ca=5 Pa	100.0	49.0	46.2	109.5	102.2
	Ca=10 Pa	100.0	47.7	45.2	109.5	102.0
	Ca=20 Pa	100.0	41.5	42.4	107.1	98.4
	Ca=30 Pa	100.0	38.0	37.4	110.8	102.4
	Ca=40 Pa	100.0	40.3	45.7	107.3	101.0
	Ca=80 Pa	100.0	42.2	40.7	110.5	102.6
	Ca=120 Pa	100.0	43.7	42.1	110.0	102.4
Y(NO)	Ca=5 Pa	100.0	207.4	213.1	83.8	98.1
	Ca=10 Pa	100.0	210.0	214.7	83.8	98.2
	Ca=20 Pa	100.0	224.7	222.2	89.2	106.6
	Ca=30 Pa	100.0	221.9	222.2	83.6	99.4
	Ca=40 Pa	100.0	235.4	223.3	89.1	101.7
	Ca=80 Pa	100.0	220.6	222.1	84.2	99.9
	Ca=120 Pa	100.0	218.6	221.0	83.9	99.2

Table S4. The response of CO_2 assimilation and chlorophyll fluorescence parameters to changes in light intensity (A:Q curve). Average, SE, 95% confidence intervals (95% CI) and percentages relative to the control (% of Control) of measurement data are presented (n=3-5, except for After Day 8 of Control, where n=1, because two trees lost the marked leaf by defoliation or dead). The same data as in Figure A2 were used.

Average

		4-Dec-23	Day 7 (18/Dec/23)		After day 8 (26/Dec/23)	
		Unstressed	Heat	Control	Heat	Control
CO_2 assimilation rate	0 μmol m⁻² s⁻¹	-0.169	-0.614	-0.793	-0.125	-0.227
	50 μmol m⁻² s⁻¹	0.780	-0.153	-0.262	0.629	0.548
	100 μmol m⁻² s⁻¹	1.275	-0.048	-0.142	0.777	0.892
	300 μmol m⁻² s⁻¹	1.781	0.034	-0.149	0.974	1.237
	500 μmol m⁻² s⁻¹	2.171	0.107	-0.065	1.040	1.509
	700 μmol m⁻² s⁻¹	2.470	0.075	-0.018	1.112	1.584
	1,000 μmol m⁻² s⁻¹	2.600	0.076	-0.069	1.062	1.478
	1,500 μmol m⁻² s⁻¹	2.734	0.279	0.015	1.463	0.790
Electron transport rate	0 μmol m⁻² s⁻¹	-	-	-	-	-
	50 μmol m⁻² s⁻¹	8.513	2.643	2.478	6.868	8.267
	100 μmol m⁻² s⁻¹	13.204	3.909	3.693	9.558	12.192
	300 μmol m⁻² s⁻¹	18.805	5.671	5.370	12.757	16.472
	500 μmol m⁻² s⁻¹	20.883	6.048	6.267	14.045	18.523
	700 μmol m⁻² s⁻¹	21.877	7.429	7.412	14.048	18.535
	1,000 μmol m⁻² s⁻¹	22.724	8.442	9.110	14.768	18.906
	1,500 μmol m⁻² s⁻¹	24.793	10.695	12.001	17.080	18.977
1-qL	0 μmol m⁻² s⁻¹	-	-	-	-	-
	50 μmol m⁻² s⁻¹	0.285	0.370	0.446	0.241	0.225
	100 μmol m⁻² s⁻¹	0.370	0.544	0.596	0.354	0.322
	300 μmol m⁻² s⁻¹	0.623	0.783	0.794	0.655	0.629
	500 μmol m⁻² s⁻¹	0.744	0.858	0.841	0.770	0.743
	700 μmol m⁻² s⁻¹	0.808	0.873	0.861	0.835	0.815
	1,000 μmol m⁻² s⁻¹	0.861	0.897	0.869	0.877	0.867
	1,500 μmol m⁻² s⁻¹	0.901	0.941	0.883	0.920	0.924
Y(II)	0 μmol m⁻² s⁻¹	-	-	-	-	-
	50 μmol m⁻² s⁻¹	0.404	0.125	0.117	0.326	0.393
	100 μmol m⁻² s⁻¹	0.313	0.093	0.088	0.227	0.289
	300 μmol m⁻² s⁻¹	0.149	0.045	0.042	0.101	0.130
	500 μmol m⁻² s⁻¹	0.099	0.029	0.030	0.067	0.088
	700 μmol m⁻² s⁻¹	0.074	0.025	0.025	0.048	0.063
	1,000 μmol m⁻² s⁻¹	0.054	0.020	0.022	0.035	0.045
	1,500 μmol m⁻² s⁻¹	0.039	0.017	0.019	0.027	0.030
Y(NPQ)	0 μmol m⁻² s⁻¹	-	-	-	-	-
	50 μmol m⁻² s⁻¹	0.331	0.368	0.302	0.418	0.324
	100 μmol m⁻² s⁻¹	0.435	0.380	0.312	0.549	0.458
	300 μmol m⁻² s⁻¹	0.596	0.386	0.320	0.672	0.609
	500 μmol m⁻² s⁻¹	0.629	0.390	0.323	0.696	0.640
	700 μmol m⁻² s⁻¹	0.643	0.388	0.323	0.710	0.657
	1,000 μmol m⁻² s⁻¹	0.653	0.387	0.323	0.718	0.668
	1,500 μmol m⁻² s⁻¹	0.658	0.250	0.298	0.700	0.659
Y(NO)	0 μmol m⁻² s⁻¹	-	-	-	-	-
	50 μmol m⁻² s⁻¹	0.265	0.506	0.581	0.255	0.283
	100 μmol m⁻² s⁻¹	0.251	0.528	0.601	0.225	0.253
	300 μmol m⁻² s⁻¹	0.255	0.569	0.637	0.228	0.260
	500 μmol m⁻² s⁻¹	0.272	0.581	0.647	0.237	0.272
	700 μmol m⁻² s⁻¹	0.283	0.587	0.651	0.242	0.280
	1,000 μmol m⁻² s⁻¹	0.293	0.593	0.655	0.247	0.287
	1,500 μmol m⁻² s⁻¹	0.302	0.733	0.683	0.273	0.311

Table S4 continued. **SE**

		4-Dec-23	Day 7 (18/Dec/23)		After day 8 (26/Dec/23)	
		Unstressed	Heat	Control	Heat	Control
CO_2 assimilation rate	0 μmol m^{-2} s^{-1}	0.033	0.041	0.070	0.030	-
	50 μmol m^{-2} s^{-1}	0.146	0.081	0.277	0.341	-
	100 μmol m^{-2} s^{-1}	0.235	0.006	0.318	0.459	-
	300 μmol m^{-2} s^{-1}	0.268	0.056	0.329	0.497	-
	500 μmol m^{-2} s^{-1}	0.246	0.065	0.344	0.478	-
	700 μmol m^{-2} s^{-1}	0.223	0.072	0.319	0.432	-
	1,000 μmol m^{-2} s^{-1}	0.274	0.033	0.319	0.470	-
	1,500 μmol m^{-2} s^{-1}	0.362	0.112	0.343	0.083	-
Electron transport rate	0 μmol m^{-2} s^{-1}	-	-	-	-	-
	50 μmol m^{-2} s^{-1}	0.590	0.759	1.044	1.337	-
	100 μmol m^{-2} s^{-1}	0.765	1.123	1.600	1.579	-
	300 μmol m^{-2} s^{-1}	0.713	1.285	2.039	1.898	-
	500 μmol m^{-2} s^{-1}	0.928	1.319	2.074	1.818	-
	700 μmol m^{-2} s^{-1}	1.026	1.065	2.284	2.376	-
	1,000 μmol m^{-2} s^{-1}	1.006	1.003	1.848	2.184	-
	1,500 μmol m^{-2} s^{-1}	0.876	1.471	1.675	3.918	-
1-qL	0 μmol m^{-2} s^{-1}	-	-	-	-	-
	50 μmol m^{-2} s^{-1}	0.037	0.054	0.100	0.011	-
	100 μmol m^{-2} s^{-1}	0.022	0.035	0.073	0.008	-
	300 μmol m^{-2} s^{-1}	0.026	0.001	0.016	0.015	-
	500 μmol m^{-2} s^{-1}	0.021	0.006	0.029	0.013	-
	700 μmol m^{-2} s^{-1}	0.016	0.012	0.031	0.011	-
	1,000 μmol m^{-2} s^{-1}	0.012	0.012	0.042	0.011	-
	1,500 μmol m^{-2} s^{-1}	0.009	0.005	0.049	0.016	-
Y(II)	0 μmol m^{-2} s^{-1}	-	-	-	-	-
	50 μmol m^{-2} s^{-1}	0.028	0.036	0.049	0.063	-
	100 μmol m^{-2} s^{-1}	0.018	0.027	0.038	0.038	-
	300 μmol m^{-2} s^{-1}	0.006	0.010	0.016	0.015	-
	500 μmol m^{-2} s^{-1}	0.004	0.006	0.010	0.009	-
	700 μmol m^{-2} s^{-1}	0.003	0.004	0.008	0.008	-
	1,000 μmol m^{-2} s^{-1}	0.002	0.002	0.004	0.005	-
	1,500 μmol m^{-2} s^{-1}	0.001	0.002	0.003	0.006	-
Y(NPQ)	0 μmol m^{-2} s^{-1}	-	-	-	-	-
	50 μmol m^{-2} s^{-1}	0.022	0.016	0.031	0.084	-
	100 μmol m^{-2} s^{-1}	0.016	0.013	0.035	0.050	-
	300 μmol m^{-2} s^{-1}	0.009	0.013	0.038	0.030	-
	500 μmol m^{-2} s^{-1}	0.011	0.016	0.039	0.025	-
	700 μmol m^{-2} s^{-1}	0.012	0.015	0.039	0.025	-
	1,000 μmol m^{-2} s^{-1}	0.013	0.016	0.040	0.023	-
	1,500 μmol m^{-2} s^{-1}	0.013	0.057	0.041	0.008	-
Y(NO)	0 μmol m^{-2} s^{-1}	-	-	-	-	-
	50 μmol m^{-2} s^{-1}	0.013	0.028	0.080	0.022	-
	100 μmol m^{-2} s^{-1}	0.011	0.025	0.072	0.013	-
	300 μmol m^{-2} s^{-1}	0.011	0.017	0.053	0.016	-
	500 μmol m^{-2} s^{-1}	0.013	0.016	0.048	0.017	-
	700 μmol m^{-2} s^{-1}	0.013	0.016	0.045	0.017	-
	1,000 μmol m^{-2} s^{-1}	0.013	0.017	0.044	0.018	-
	1,500 μmol m^{-2} s^{-1}	0.013	0.058	0.044	0.005	-

Table S4 continued. **95% CI**

		4-Dec-23	Day 7 (18/Dec/23)		After day 8 (26/Dec/23)	
		Unstressed	Heat	Control	Heat	Control
CO_2 assimilation rate	0 μmol m^{-2} s^{-1}	0.064	0.081	0.138	0.059	-
	50 μmol m^{-2} s^{-1}	0.286	0.158	0.543	0.668	-
	100 μmol m^{-2} s^{-1}	0.461	0.012	0.623	0.899	-
	300 μmol m^{-2} s^{-1}	0.526	0.110	0.645	0.974	-
	500 μmol m^{-2} s^{-1}	0.482	0.128	0.673	0.937	-
	700 μmol m^{-2} s^{-1}	0.438	0.141	0.626	0.847	-
	1,000 μmol m^{-2} s^{-1}	0.538	0.066	0.626	0.922	-
	1,500 μmol m^{-2} s^{-1}	0.710	0.220	0.673	0.162	-
Electron transport rate	0 μmol m^{-2} s^{-1}	-	-	-	-	-
	50 μmol m^{-2} s^{-1}	1.157	1.488	2.047	2.621	-
	100 μmol m^{-2} s^{-1}	1.500	2.201	3.136	3.095	-
	300 μmol m^{-2} s^{-1}	1.397	2.519	3.996	3.719	-
	500 μmol m^{-2} s^{-1}	1.820	2.585	4.065	3.563	-
	700 μmol m^{-2} s^{-1}	2.012	2.087	4.477	4.657	-
	1,000 μmol m^{-2} s^{-1}	1.971	1.966	3.622	4.281	-
	1,500 μmol m^{-2} s^{-1}	1.718	2.882	3.283	7.679	-
1-qL	0 μmol m^{-2} s^{-1}	-	-	-	-	-
	50 μmol m^{-2} s^{-1}	0.072	0.106	0.197	0.022	-
	100 μmol m^{-2} s^{-1}	0.043	0.069	0.142	0.016	-
	300 μmol m^{-2} s^{-1}	0.050	0.002	0.031	0.029	-
	500 μmol m^{-2} s^{-1}	0.041	0.012	0.057	0.026	-
	700 μmol m^{-2} s^{-1}	0.032	0.023	0.062	0.022	-
	1,000 μmol m^{-2} s^{-1}	0.024	0.024	0.082	0.022	-
	1,500 μmol m^{-2} s^{-1}	0.018	0.010	0.096	0.032	-
Y(II)	0 μmol m^{-2} s^{-1}	-	-	-	-	-
	50 μmol m^{-2} s^{-1}	0.055	0.071	0.097	0.124	-
	100 μmol m^{-2} s^{-1}	0.035	0.052	0.075	0.074	-
	300 μmol m^{-2} s^{-1}	0.011	0.020	0.032	0.029	-
	500 μmol m^{-2} s^{-1}	0.009	0.012	0.019	0.017	-
	700 μmol m^{-2} s^{-1}	0.007	0.007	0.015	0.016	-
	1,000 μmol m^{-2} s^{-1}	0.005	0.005	0.009	0.010	-
	1,500 μmol m^{-2} s^{-1}	0.003	0.005	0.005	0.012	-
Y(NPQ)	0 μmol m^{-2} s^{-1}	-	-	-	-	-
	50 μmol m^{-2} s^{-1}	0.044	0.031	0.061	0.164	-
	100 μmol m^{-2} s^{-1}	0.031	0.026	0.068	0.097	-
	300 μmol m^{-2} s^{-1}	0.017	0.026	0.074	0.059	-
	500 μmol m^{-2} s^{-1}	0.021	0.031	0.077	0.049	-
	700 μmol m^{-2} s^{-1}	0.023	0.029	0.076	0.048	-
	1,000 μmol m^{-2} s^{-1}	0.025	0.031	0.079	0.044	-
	1,500 μmol m^{-2} s^{-1}	0.025	0.111	0.081	0.016	-
Y(NO)	0 μmol m^{-2} s^{-1}	-	-	-	-	-
	50 μmol m^{-2} s^{-1}	0.025	0.055	0.157	0.043	-
	100 μmol m^{-2} s^{-1}	0.022	0.050	0.142	0.025	-
	300 μmol m^{-2} s^{-1}	0.022	0.033	0.104	0.031	-
	500 μmol m^{-2} s^{-1}	0.025	0.032	0.094	0.033	-
	700 μmol m^{-2} s^{-1}	0.026	0.032	0.089	0.034	-
	1,000 μmol m^{-2} s^{-1}	0.026	0.033	0.086	0.036	-
	1,500 μmol m^{-2} s^{-1}	0.025	0.113	0.086	0.009	-

Table S4 continued. **% of Control**

		4-Dec-23	Day 7 (18/Dec/23)		After day 8 (26/Dec/23)	
		Unstressed	Heat	Control	Heat	Control
CO_2 assimilation rate	0 μmol m^{-2} s^{-1}	100.0	364.4	470.3	73.9	134.5
	50 μmol m^{-2} s^{-1}	100.0	-19.6	-33.6	80.7	70.2
	100 μmol m^{-2} s^{-1}	100.0	-3.8	-11.1	61.0	70.0
	300 μmol m^{-2} s^{-1}	100.0	1.9	-8.4	54.7	69.5
	500 μmol m^{-2} s^{-1}	100.0	4.9	-3.0	47.9	69.5
	700 μmol m^{-2} s^{-1}	100.0	3.0	-0.7	45.0	64.2
	1,000 μmol m^{-2} s^{-1}	100.0	2.9	-2.7	40.8	56.8
	1,500 μmol m^{-2} s^{-1}	100.0	10.2	0.5	53.5	28.9
Electron transport rate	0 μmol m^{-2} s^{-1}	-	-	-	-	-
	50 μmol m^{-2} s^{-1}	100.0	31.0	29.1	80.7	97.1
	100 μmol m^{-2} s^{-1}	100.0	29.6	28.0	72.4	92.3
	300 μmol m^{-2} s^{-1}	100.0	30.2	28.6	67.8	87.6
	500 μmol m^{-2} s^{-1}	100.0	29.0	30.0	67.3	88.7
	700 μmol m^{-2} s^{-1}	100.0	34.0	33.9	64.2	84.7
	1,000 μmol m^{-2} s^{-1}	100.0	37.1	40.1	65.0	83.2
	1,500 μmol m^{-2} s^{-1}	100.0	43.1	48.4	68.9	76.5
1-qL	0 μmol m^{-2} s^{-1}	-	-	-	-	-
	50 μmol m^{-2} s^{-1}	100.0	129.8	156.4	84.6	79.0
	100 μmol m^{-2} s^{-1}	100.0	147.0	161.3	95.6	87.0
	300 μmol m^{-2} s^{-1}	100.0	125.7	127.4	105.1	100.9
	500 μmol m^{-2} s^{-1}	100.0	115.4	113.1	103.5	100.0
	700 μmol m^{-2} s^{-1}	100.0	108.0	106.5	103.4	100.9
	1,000 μmol m^{-2} s^{-1}	100.0	104.2	100.9	101.8	100.7
	1,500 μmol m^{-2} s^{-1}	100.0	104.5	98.1	102.1	102.6
Y(II)	0 μmol m^{-2} s^{-1}	-	-	-	-	-
	50 μmol m^{-2} s^{-1}	100.0	31.0	29.1	80.7	97.3
	100 μmol m^{-2} s^{-1}	100.0	29.6	28.0	72.4	92.2
	300 μmol m^{-2} s^{-1}	100.0	30.1	28.6	67.8	87.6
	500 μmol m^{-2} s^{-1}	100.0	29.0	30.0	67.2	88.7
	700 μmol m^{-2} s^{-1}	100.0	34.0	33.9	64.2	84.7
	1,000 μmol m^{-2} s^{-1}	100.0	37.1	40.1	65.0	83.2
	1,500 μmol m^{-2} s^{-1}	100.0	43.1	48.4	68.9	76.5
Y(NPQ)	0 μmol m^{-2} s^{-1}	-	-	-	-	-
	50 μmol m^{-2} s^{-1}	100.0	111.3	91.2	126.5	98.1
	100 μmol m^{-2} s^{-1}	100.0	87.2	71.6	126.0	105.3
	300 μmol m^{-2} s^{-1}	100.0	64.8	53.7	112.7	102.3
	500 μmol m^{-2} s^{-1}	100.0	62.1	51.4	110.8	101.8
	700 μmol m^{-2} s^{-1}	100.0	60.3	50.3	110.5	102.2
	1,000 μmol m^{-2} s^{-1}	100.0	59.2	49.5	110.0	102.4
	1,500 μmol m^{-2} s^{-1}	100.0	37.9	45.3	106.3	100.1
Y(NO)	0 μmol m^{-2} s^{-1}	-	-	-	-	-
	50 μmol m^{-2} s^{-1}	100.0	190.9	218.9	96.3	106.6
	100 μmol m^{-2} s^{-1}	100.0	209.9	238.9	89.3	100.5
	300 μmol m^{-2} s^{-1}	100.0	222.8	249.8	89.2	102.0
	500 μmol m^{-2} s^{-1}	100.0	213.3	237.7	87.0	100.0
	700 μmol m^{-2} s^{-1}	100.0	207.4	230.1	85.5	99.1
	1,000 μmol m^{-2} s^{-1}	100.0	202.3	223.5	84.1	97.8
	1,500 μmol m^{-2} s^{-1}	100.0	242.6	225.9	90.2	102.9
